# Photodetection Tuning with High Absorptivity Using Stacked 2D Heterostructure Films

**DOI:** 10.3390/nano12040712

**Published:** 2022-02-21

**Authors:** Umar Farooq, Kossi A. A. Min-Dianey, Pandey Rajagopalan, Muhammad Malik, Damgou Mani Kongnine, Jeong Ryeol Choi, Phuong V. Pham

**Affiliations:** 1 Paul C. Lauterbur Research Center for Biomedical Imaging, Institute of Biomedical and Health Engineering, Shenzhen Institutes of Advanced Technology, Chinese Academy of Sciences, 1068 Xueyuan Avenue, Shenzhen 518055, China; farooq@siat.ac.cn; 2 Department of Physics, Laboratoire Sur l’Energie Solaire, Université de Lomé, Lome 01BP1515, Togo; anyaratt20@yahoo.fr (K.A.A.M.-D.); kongnine@gmail.com (D.M.K.); 3 International Joint Innovation Center, Zhejiang University, Haining 314400, China; rajagopalanpandey@gmail.com; 4 Department of Electrical Engineering and Technology, Government College University, Faisalabad 38000, Pakistan; mmalik@gcuf.edu.pk; 5 Department of Nanoengineering, Kyonggi University, Suwon 16227, Korea; 6 SKKU Advanced Institute of Nano Technology, Sungkyunkwan University, Suwon 440-746, Korea

**Keywords:** Si film, graphite film, PD device, 2D heterostructure film, responsivity, absorptivity

## Abstract

Graphene-based photodetection (PD) devices have been broadly studied for their broadband absorption, high carrier mobility, and mechanical flexibility. Owing to graphene’s low optical absorption, the research on graphene-based PD devices so far has relied on hybrid heterostructure devices to enhance photo-absorption. Designing a new generation of PD devices supported by silicon (Si) film is considered as an innovative technique for PD devices; Si film-based devices are typically utilized in optical communication and image sensing owing to the remarkable features of Si, e.g., high absorption, high carrier mobility, outstanding CMOS integration. Here, we integrate (i) Si film via a splitting/printing transfer with (ii) graphite film grown by a pyrolysis method. Consequently, p-type Si film/graphite film/n-type Si-stacked PD devices exhibited a broadband detection of 0.4–4 μm (in computation) and obtained good experimental results such as the responsivity of 100 mA/W, specific detectivity of 3.44 × 10^6^ Jones, noise-equivalent power of 14.53 × 10^−10^ W/(Hz)^1/2^, external quantum efficiency of 0.2, and rise/fall time of 38 μs/1 μs under 532 nm laser illumination. Additionally, our computational results also confirmed an enhanced light absorption of the above stacked 2D heterostructure film-based PD device compatible with the experimental results.

## 1. Introduction

The history of photodetection (PD) devices has been established [1], and a wide range of graphene-related applications have been indicated and are expected in the near future [2]. Silicon (Si)-based high-performance electronic sand optoelectronics such as graphene-Si PD devices suffer from very low responsivity, mainly due to the low optical absorption of graphene (~2.3%). Many efforts to improve the responsivity have been reported, such as using an optical resonance micro-cavity integrated graphene [3], or using colloidal quantum dots covered with mono- or bilayer graphene [4]; however, the efficiencies of these devices are still not high. In another report, a heterojunction PD shows some difficulty concerning switching, limiting its use in broadband communication [5]. The ideal strategy to achieve high responsivity without sacrificing the bandwidth would be to enhance the electric field. Among these proposed concepts, a metal-grating detector shows promise in responsivity, along with the potential in broad-spectrum operation as well as spectral selectivity [6]. Hence, graphene/Si heterojunction should be used for a variety of tunable optoelectronic devices with high responsivity over a broad spectral bandwidth in the visible region. Their high responses and low dark currents render them with a high switching ratio and low dark-power consumption.

The huge potential of two-dimensional (2D) materials (hBN, MoS_2_, black phosphorous, and graphite (multilayer graphene)) for manufacturing the detecting devices covering broadband concerning spectra have been indicated and analyzed concretely [7]. Graphene with linear energy dispersion and weak electron–phonon interaction is highly anticipated to harvest hot-electrons in a broad wavelength range from ultraviolet to terahertz [8,9]. However, the limited absorption and serious backscattering of hot-electrons associated with single-layer graphene result in inadequate quantum yields [10,11,12], impeding their practically broadband PD, especially in the mid-infrared (MIR) and far-infrared (FIR) range. Schottky junction, a kind of metal/semiconductor heterojunction, plays an important role in modern optoelectronics for high-speed optical communications and PD in the visible and near-infrared (NIR) range [13,14]. In the MIR regime, the traditional metal-based Schottky junctions show high noise and low quantum yield at room temperature because of the non-ideal interface (Fermi-level pining) and strong electron–phonon coupling in metal (~0.1 ps of carrier relaxation time) [11,15], posing a big challenge for developing an alternative metallic layer. The emerging zero-bandgap graphene can probably improve the junction interface and suppress the device noise due to its ultra-fast electron–electron interaction [11,15], improved electron–phonon coupling (~1 ps of carrier relaxation time), dangling-bond free surface, and van der Waals contact with semiconductors [16]. However, the high-crystalline graphene, with dominant single layers, has only 2.3% light absorption, which is too low for MIR PD.

Recently, Si-on-insulator or Si membrane has been emerging as a promising semiconductor to expectedly enhance the fast response, high responsivity, and specific detectivity for PD devices [17]. Si substrate is an optimal choice in photonics due to its potential for integrating optics with electronics from micro- to macro-scale on a single chip [2,7,16,18,19,20,21]. While Si-based PDs have been developed for commercial uses, intrinsic Si is not a broadband optical material, though its absorption edge can be tuned by doping. Other drawbacks of Si include low light emission and electro-optic efficiency due to its indirect bandgap. Graphene, a 2D carbon sheet, is rapidly emerging for photonics and electronics. Graphite and graphene show superior properties over Si and are considered as potential material for a next-generation semiconductor and display devices to replace currently limited device technologies [21,22,23,24,25,26,27,28,29,30,31,32,33,34,35,36,37]. In addition, graphite and graphene have also exhibited mechanical flexibility and transparency, which are highly desirable for wearable devices or detection applications [21,22,23,24,25,26,27,28,29,30,31,32,33,34,35,36,37]. Compared to other 2D materials, graphene is much more mature for large-scale mass production. One of the important and attractive aspects of nanostructures lies in using graphene material as a nano-scale thin film for heterostructure devices.

Here, we utilize a graphite film (~15 nm thick, ~40 layers) to tune the PD wavelength of Si-based Schottky diode to 4 μm. We propose a feasible way to develop low-cost and large-scale broadband graphite film-based PD devices for CMOS image sensors at room temperature by stacking p-type Si film with graphite film located on n-type Si substrate. Two benefits of our micro–nano fabrication process are that (i) micro/nanostructure increases the photon absorption by adding more surface state for converting photons to electrons, and (ii) provides stable contact between Si film and graphite film. As a result, our device performances such as photoresponsivity, detectivity, and rise/fall time in the broader bandwidth of the wavelength were enhanced. It is observed that the photodiode is sensitive to visible light at broadband spectra (0.4–4 μm), with relatively high responsivity of 100 mA/W, specific detectivity of 3.44 × 10^6^ Jones, noise-equivalent power of 14.53 × 10^−10^ W/(Hz)^1/2^, external quantum efficiency of 0.2, and a high response rate with rise time of 38 μs and fall time of 1 μs under 532 nm laser illumination. This work unveils the potential of Si film/graphite film/Si Schottky diode for broadband optoelectronics and image sensors with high stability in ambient environment. 

## 2. Materials, Devices, and Methods

Material Preparation of Thin Films: (i) For graphite film: The intrinsic graphite film (~15 nm thick, ~40 layers) was manufactured similarly to the previous reports by Sone et al. [38] through the exfoliation of a highly oriented pyrolytic nanofilm crystal micro-sheet. (ii) For Si film: Si film (3 μm thick) was transferred onto graphite film/Si substrate through an integrated splitting/printing process supported by a polydimethylsiloxane (PDMS) layer.

**Figure 1:**
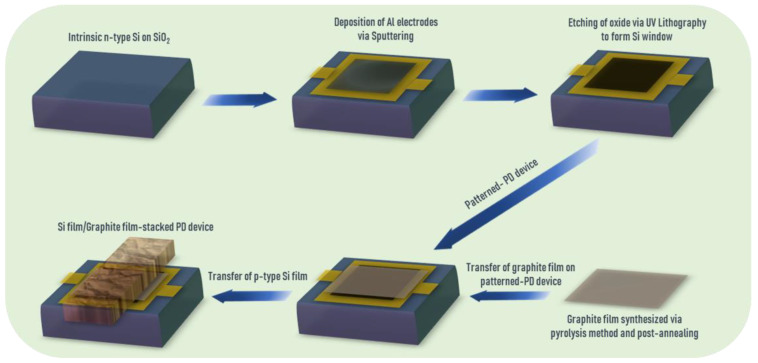
Fabrication process of Si film/graphite film-stacked photodetection (PD) devices.

Device Processing: The PD devices are manufactured on a SiO_2_ layer. (i) The SiO_2_ layer was patterned by UV lithography. Electron-beam deposition processes are utilized to deposit Au electrodes (60 nm thick) as contact pads onto SiO_2_. (ii) Photolithography was utilized to pattern the window (5 × 5 mm^2^). Then, SiO_2_ in the window is etched away by a buffered oxide etchant to form a Si window. (iii) To form a graphite film/Si Schottky junction, the graphite film was transferred to the top of the etched Si window. (iv) Ohmic contact is formed by using GaIn paste on the backside of the SiO_2_ substrate and finally, Au wire is bonded to the top electrodes. Finally, a p-type Si film (3 μm thick) was transferred onto graphite film/n-type Si-stacked PD device (Figure 1).

Characterization: A focused ion beam (FIB, Helios 450HP, Thermofisher Scientific Company, Waltham, MA, USA) was used to prepare cross-sectional and top-view imaging of Si film/graphite film/Si-stacked PD devices. Energy-dispersive X-ray spectroscopy (EDS, FEI Verios 460, 1 kV, Thermofisher Scientific Comp., Waltham, MA, USA) was used for mapping and imaging of Si, O, and C elements of PD devices based on graphite film/Si and Si film/graphite film/Si. I–V curves are characterized with the Agilent Semiconductor Analyzer (B1500, Keysight Technology Company, Santa Rosa, CA, USA) using a 532 nm wavelength for the Si film/graphite film/Si-stacked PD devices. UV–Vis spectra (Shimadzu-3600, Shimadzu Corp., Tokyo, Japan) is utilized to measure the optical transparency of the device. Raman spectra (RM-1000 Invia, Renishaw plc., Wolton-under-Edge, Gloucestershire, UK) is utilized for the analysis of graphite film. A transmission electron microscope (TEM, FEI Titan 80/300, FEI Company, Hillsboro, OR, USA) was used to observe the surface morphology of the intrinsic graphite film.

## 3. Results and Discussion

**Figure 2:**
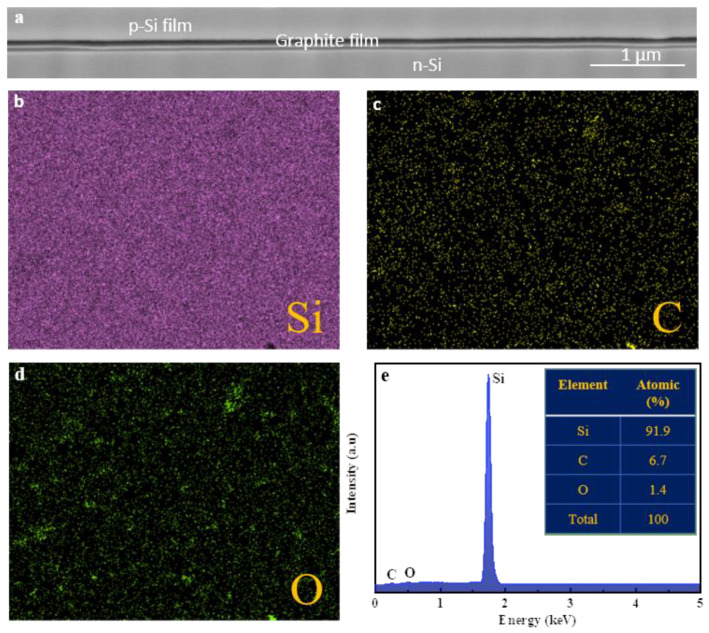
(**a**) Focus-ion beam (FIB) image, and (**b**–**e**) Energy-dispersive X-ray spectroscopy (EDS) mapping and images of Si, C, and O elements of Si film/graphite film/Si-stacked PD device.

Figure 1 depicts the fabrication process of p-type Si film/graphite nanofilm/n-type Si-stacked PD device (see the detail in Section 2). Here, intrinsic graphite film showed high light transparency (80.64%) on glass at 532 nm visible wavelength (Supplementary Materials Figure S1). In addition, Raman data revealed the multilayer phase of intrinsic graphite film (Figure S2) with ~40 atomic layers through a cross-sectional TEM image (Figure S3). Here, the thickness of this graphite film is estimated through *d_0_* = 0.335 nm spacing from TEM measurement formed and holed by weak van der Waals force [39]. To visualize the morphology, element compositions, and absorptivity of PD devices stacked by p-type Si film/graphite film/n-type Si heterostructure, the FIB, EDS, and FTIR spectroscopies were taken (Figure 2). Figure S4 and Figure 2a revealed the top-view and cross-sectional FIB images with the good interface contacts of this heterostructure. Meanwhile, Figure 2c–e exhibit the element mapping of Si, C, and O of this heterostructure corresponding with the atomic percentages of 91.9%, 6.7%, and 1.4% at high uniform coverage on this stacked heterostructure PD device, respectively (Figure 2b–e). Compared with the PD device without the top p-type Si film, the atomic percentages of Si and O elements were lower (33% and 0.7%, respectively) and that of C element was higher in value (66.3%) (Figure S5). Additionally, the absorptivity of a stacked 2D heterostructure of Si film/graphite film/Si exhibited a high absorptivity at λ = 0.532 µm compared to graphite film/Si structure without the top Si film, which is a great advantage for light absorption enhancement of this PD device.

**Figure 3:**
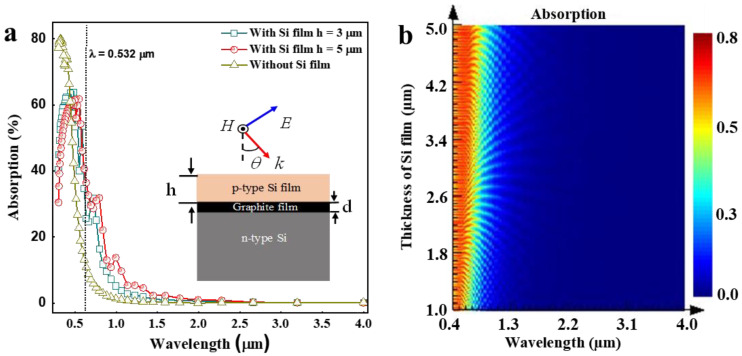
Simulated absorption characteristics through the cross-section of Si film/graphite film/Si-stacked heterostructure: (**a**) Absorption spectra, and (**b**) Absorption map as a function of the wavelength (0.4–4 µm) and the top Si film thickness range (1–5 µm) exhibiting the field distribution.

To predict the absorption enhancement, a 3D finite-difference time-domain (3D-FDTD) method was applied. A p-polarized plane wave source was injected with an incident wavelength sweep from 0.4 to 4 µm. The periodic boundary conditions were applied on the x and y-directions while the absorbing perfectly matched layers were used in the z-direction (direction of propagation). In order to achieve high accuracy computation with full coverage of the structure, a graded mesh with “conformal variant 1” was set. In addition, the optical map of the absorptivity distribution through the Si film/graphite film/Si-stacked heterostructure was performed through the optimization and sweeps scripting. With the satisfactory convergence criterion, the absorption spectra A(λ) was computed from the reflected and transmitted fields through the structure by the following conservation relation A(λ) + R(λ) + T(λ) = 1, where λ is the wavelength, and R(λ) and T(λ) are the reflected and transmitted spectra, respectively. The details of the simulation process can be found in the previous report [40]. As observed in Figure 3a, the absorption exhibits a strong enhancement in the visible wavelength around λ = 0.5 µm with the integration of the top Si film on graphite film/Si-stacked heterostructure. At the wavelength λ = 0.532 µm, the absorption remains almost constant for both thicknesses h = 3 µm and h = 5 µm considerations. Thus, to satisfy the miniaturization, h = 3 µm was selected as the appropriate thickness for the top Si film to achieve the experimental design at λ = 0.532 µm, resulting in further enhancement of the device photoresponsivity. Here, in Figure 3a, the absorptivity values at the wavelength of 0.532 μm, 1 μm, and 1.77 μm without top Si film are 26.73%, 1.33%, and 0.08%; with top Si film (3 μm thick) they are 57.7%, 5.15%, and 0.83%; and with top Si film (5 μm thick) they are 59.78%, 13.72%, and 1.48%, respectively. Naturally, the Si film is an extremely crispy material and breaks very easily during the Si transfer process onto graphite film-stacked PD devices; therefore, it is very difficult to exfoliate and transfer. Indeed, in our experiment, this broken effect occurred with Si film of 5 μm thick, but did not occur with Si film 3 μm thick. The results from our FDTD simulation confirmed that the absorptivity of the thicker Si film (5 μm) is better than the thinner Si film (3 μm). However, the thicker Si film (5 μm) is an upper limit of the breaking effect. Thus, the thinner Si film (3 μm) was selected in this study. Moreover, Figure 3b shows the optical absorbed maps as a function of the wavelength range (0.4–4 µm) and the thickness of the top Si film over the given range (1–5 µm). These results provide the full prediction of the absorption in terms of near-field distribution through the Si film/graphite film/Si-stacked heterostructure and suitably allow the identification and extraction of the optimum characteristics for high absorptivity through the Si film/graphite film/Si-stacked PD device.

**Figure 4:**
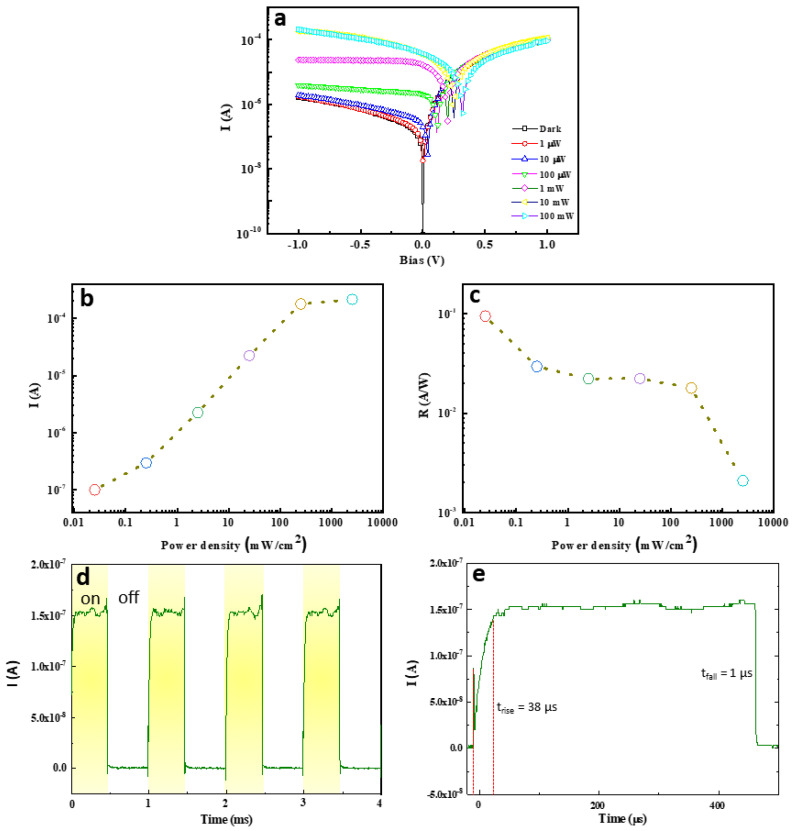
(**a**) I–V curves of Si film/graphite film-stacked PD devices under dark and laser illumination at 532 nm wavelength with different power values. (**b**,**c**) Photocurrent and photoresponsivity as the functions of power density values at bias = −1 V of Si film/graphite film-stacked PD devices, respectively. (**d**,**e**) Rise time and fall time at 3 mW, 1 kHz of Si film/graphite film-stacked PD device.

To characterize the possibility of optical sensing of Si film/graphite film/Si-stacked PD devices, we carried out the experiments under dark and laser illumination conditions at 532 nm wavelength with different power density values (Figure 4). I–V curves are utilized for this measurement assisted by the laser system setup (Figure S6). As a result, Figure 4a revealed the dark current (*I_dark_*) and the photocurrent (*I_light_*) as the functions of bias vary from −1 to 1 V, with exposure under 532 nm visible light with different incident powers (*P_incident_*) from 1 μW to 100 mW. The more the increase in the laser power intensity, the more the increase in the photocurrent also at the reverse bias (−1–0 V) (Figure 4b). In the optoelectronic field, photoresponsivity,

R = (*I*_*light*_ − *I*_*dark*_)/*P*_*incident*_, (1)

is one of the key factors in assessing the PD possibility of the device. In particular, the highest R value was achieved at 100 mA/W at a reverse bias of −1 V (Figure 4c). That is clear experimental evidence of the stacked Si film/graphite film heterostructure that enables the enhancement of the light absorption. On the other hand, the other parameters of Si film/graphite film/Si surface are also calculated such as rise/fall time (taken as 90% of the rise value and fall value), the noise-equivalent power,

(2) $$\mathrm{NEP}=\frac{\sqrt{2e\cdot I_{dark}}}{R\cdot \sqrt{A}}$$

(*i*th *A* is the effective area (5 × 5 mm^2^), the specific detectivity,

(3) $$D*=\frac{\sqrt{A}}{NEP}$$

and external quantum efficiency,

(4) $$\mathrm{EQE}=\frac{Rhc}{e\lambda }$$

where *h* is Planck constant, *c* is the speed of light, *e* is the charge on an electron, and *λ* is visible wavelength (532 nm).

Table 1 summarized the converted results in Figure 4 with the highest responsivity, NEP, D*, rise/fall time, and EQE values of Si film/graphite film/Si-stacked PD devices at the lowest power (100 nW) and at the reverse bias of −1 V under 532 nm laser illumination. As a result, it showed 100 mA/W, 14.53 × 10^−10^ W/(Hz)^1/2^, 3.44 × 10^6^ Jones, 38 μs/1 μs (at 3 mW, 1 kHz, Figure 4d,e), and 0.2, respectively. Here, the obtained values of rise/fall time are not very fast concerning meeting the industry requirement due to the imperfection contacts at the interface of top Si film/graphite film and graphite film/Si substrate during the transfer processes of graphite film and the top Si film on Si substrate and requires more improvement in our next study.

**Figure 5:**
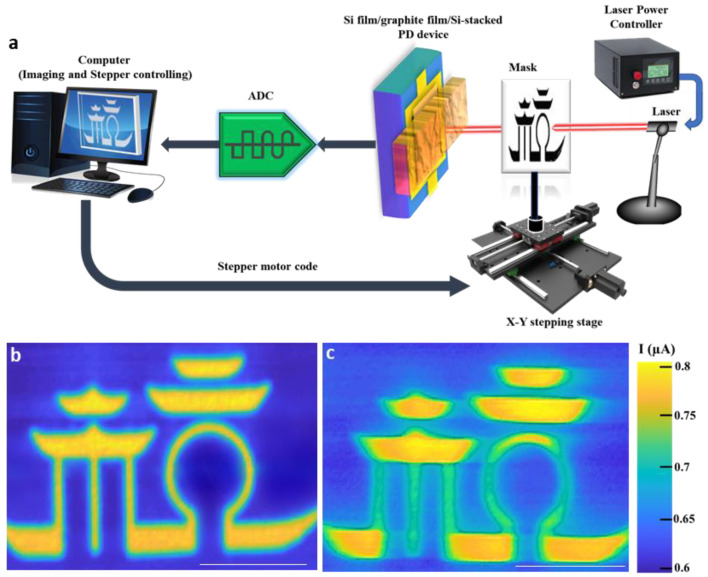
(**a**) Schematic of photo-imaging system setup using the linear array to scan image of “temple” mask pattern. Photo-imaging results of “temple” pattern under light illuminations of 0.532 μm (**b**) and 1.85 μm (**c**) for Si film/graphite film/Si-stacked Vis-IR PD devices. Scale bar, 2 mm.

To confirm the absorption enhancement in our experiment results, the photo imaging of the Si film/graphite film/Si-stacked PD device was taken through the designed photo-imaging system setup as shown in Figure 5a. Here, the photo imaging was obtained with a scanning time of 8 h and a step length of 50 μm for the “temple” mask pattern size of 6 × 9 mm^2^. As a result, the photo imaging exhibited a clearer image under 0.532 μm laser illumination compared with the one under 1.85 μm laser illumination, respectively (Figure 5b,c). These results are highly compatible with our FDTD simulation data with absorption values of 57.7% at 532 μm visible wavelength and 0.83% at 1.77 μm IR wavelength of Si film (3 μm thick)/graphite film/Si heterostructure.

**Figure 6:**
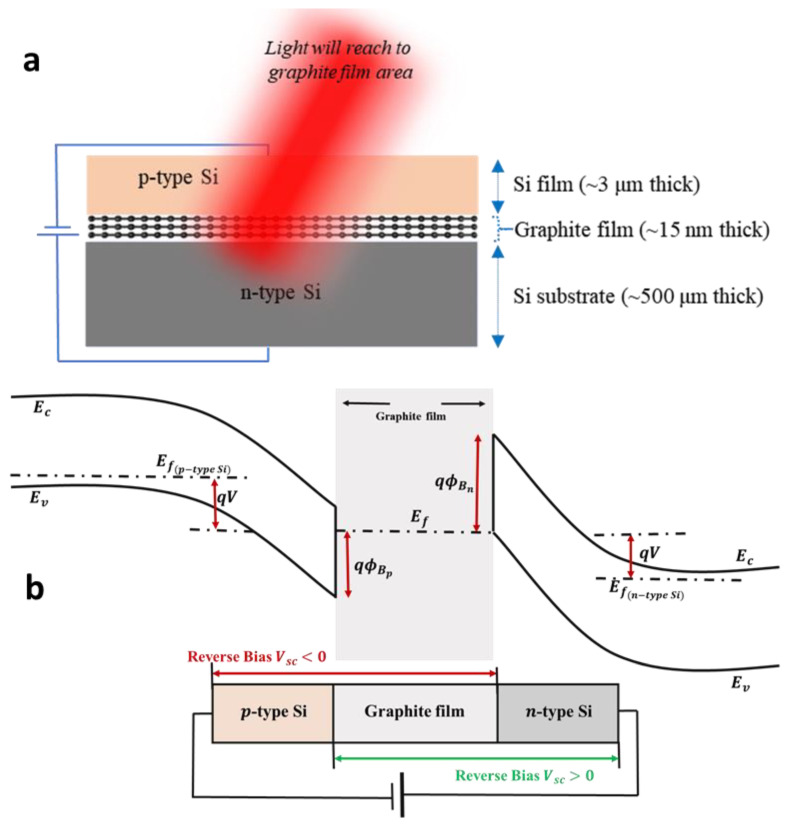
(**a**) Cross-section view of Si film/graphite film/Si-based PD device. (**b**) PD mechanism via energy band diagram structure of the broadband Vis-IR light absorption of Si film/graphite/Si Schottky photodiode junction at 532 nm laser illumination.

For the explanation of the PD mechanism of this 2D heterostructure, graphite film sandwiched between p- and n-type Si forms Schottky junctions at both interfaces (Figure 6). Actually, the Schottky junction on top Si film/CVD graphene/Ge has been reported previously by Liu et al. [17] and is rather similar to our structure. In this study, the dual Schottky junctions significantly suppress the dark current in the system, especially in the reverse bias. At low reverse bias condition of p-type Si film/graphite film/n-type Si-stacked PD device, both p-type Si film/graphite film and graphite film/n-type Si junctions are in reverse bias. As the charge transport in graphite film is mainly electron dominated, the graphite film/n-Si junction plays a crucial role in suppressing the dark current in the reverse bias. The electrons injected from p-type Si/graphite film experience a Schottky barrier of ~0.5 eV at graphite film/n-type Si interface under the dark condition. When the light with a wavelength lower than the bandgap of Si is illuminated on the device, the light absorption mainly happens in the graphite film resulting in photothermionic emission at the graphite film/n-type Si Schottky junction. In contrast, when the device is forward biased, the photoresponse is weak in the low-power region as the hole transport is substantially suppressed in graphite film which is clearly depicted in the I-V characteristics of the PD device.

## 4. Conclusions

This work demonstrates a high-performance Si film/graphite film/Si Schottky diode with high absorptivity, a fast response rate, high specific detectivity, and good response wavelength. Such outstanding detection capability is achieved by introducing Si film/graphite film that is compatible well with CMOS technology as a hybrid absorption layer, which is integrated with n-type Si substrate with a stable contact interface. It is observed that the photodiode is sensitive to visible light, with relatively high R of 100 mA/W, D* of 3.44 × 10^6^ Jones, NEP of 14.53 × 10^−10^ W/(Hz)^1/2^, rise/fall time of 38 μs/1 μs, and EQE of 0.2 under 532 nm laser illumination. In particular, regarding the simulated prediction of this 2D heterostructure. This work unveils the potential of highly stacked 2D heterostructure systems using Si film/graphite film/Si for broadband optoelectronics and image sensors with good stability in the ambient environment.

## Figures and Tables

**Table 1:** The merit of photoresponsivity (R), noise equivalent power (NEP), detectivity (D*), rise/fall time, and external quantum efficiency (EQE) values at the lowest power (100 mW) and bias (−1 V) of p-type Si film/graphite film/n-type Si-stacked PD device under 532 nm laser illumination.

	R	NEP	D*	Rise/Fall Time	EQE
Si film/graphite film/Si-stacked PD device	100 mA/W	14.53 × 10^−10^ W/(Hz)^1/2^	3.44 × 10^6^ Jones	38 μs/1 μs	0.2

## Data Availability

Not applicable.

## Supplementary Materials

The following supporting information can be downloaded at: https://www.mdpi.com/article/10.3390/nano12040712/s1, Figure S1: light transparency of graphite film; Figure S2: Raman spectra of graphite film; Figure S3: Cross-sectional TEM image of graphite film; Figure S4: top-view FIB image of Si film/graphite film/Si-stacked PD device; Figure S5: EDS mapping and images of Si, C, and O elements of graphite film on n-type Si substrate; Figure S6: Schematic of the laser system setup.
