# Supplementary material for: Photodetection Tuning with High Absorptivity Using Stacked 2D Heterostructure Films
Source: Nanomaterials (Basel). 2022 Feb 21;12(4):712. doi: 10.3390/nano12040712 (PMC8876593; doi:10.3390/nano12040712)
Supplement: Supplementary file 1 [file nanomaterials-12-00712-s001.zip › nanomaterials-1566637-supplementary.pdf]

Supporting Information

Photodetection Tuning with High Absorptivity Using Stacked 2D Heterostructure Films

Umar Farooq [1,†], Kossi A. A. Min-Dianey [2,†], Pandey Rajagopalan [3,†], Muhammad Malik [4,†], Damgou Mani Kongnine [2,†], Jeong Ryeol Choi [5,*] and Phuong V. Pham [6,*]

1 Paul C. Lauterbur Research Center for Biomedical Imaging, Institute of Biomedical and Health Engineering, Shenzhen Institutes of Advanced Technology, Chinese Academy of Sciences, 1068 Xueyuan Avenue,
Shenzhen 518055, China; farooq@siat.ac.cn

2 Department of Physics, Laboratoire Sur l'Energie Solaire, Université de Lomé, Lome 01BP1515, Togo;
anyaratt20@yahoo.fr (K.A.A.M.-D.); kongnine@gmail.com (D.M.K.)

3 International Joint Innovation Center, Zhejiang University, Haining 314400, China; rajagopalanpandey@gmail.com

4 Department of Electrical Engineering and Technology, Government College University, Faisalabad 38000, Pakistan; mmalik@gcuf.edu.pk

5 Department of Nanoengineering, Kyonggi University, Suwon 16227, Korea

6 SKKU Advanced Institute of Nano Technology, Sungkyunkwan University, Suwon 440-746, Korea

* Correspondence: jrchoi@kyonggi.ac.kr (J.R.C.); pvphuong@skku.edu (P.V.P.)

† These authors contributed equally to this work.

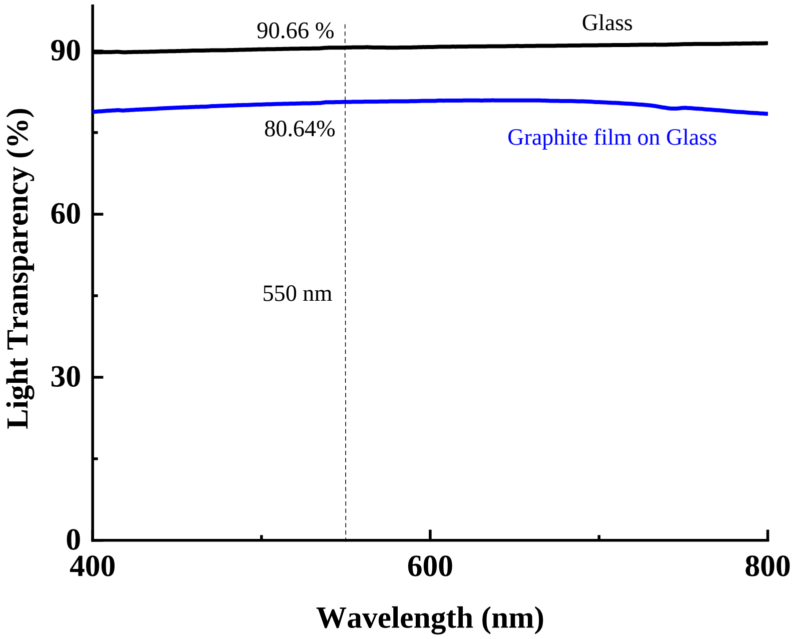

Figure S1. Light transparency of glass and intrinsic graphite film (15 nm thick)/glass.

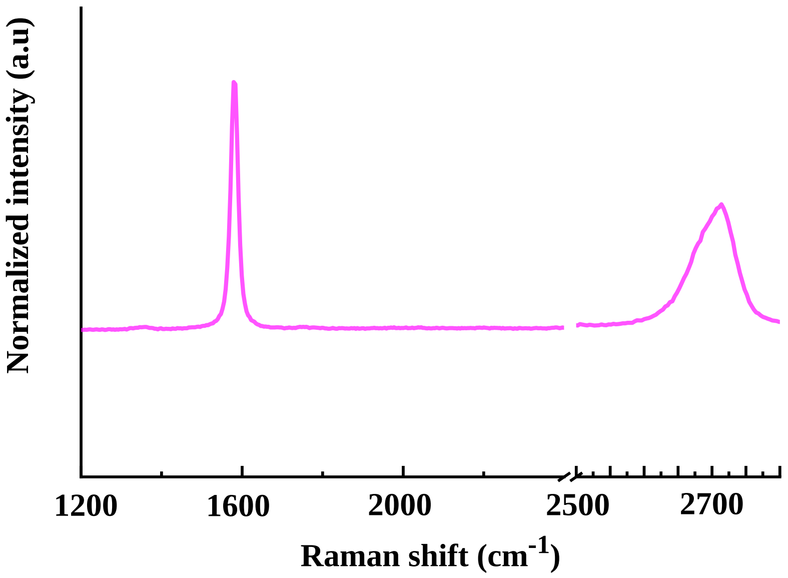

Figure S2. Raman spectra of intrinsic graphite film (15 nm thick).

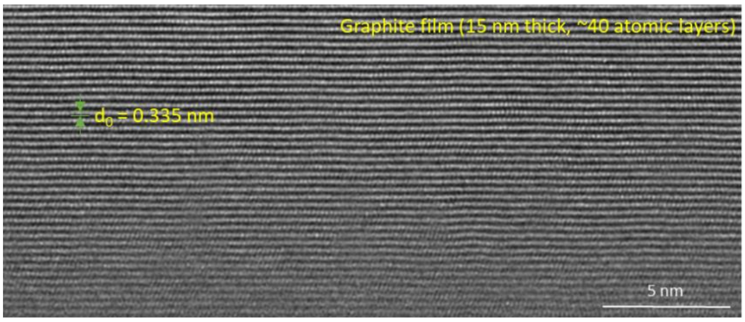

Figure S3. Cross-sectional TEM image of intrinsic graphite film (15 nm thick, >40 atomic layers).

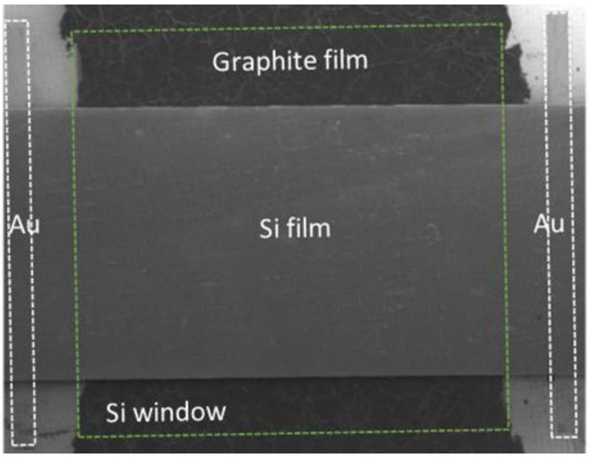

Figure S4. SEM image of Si film/graphite film/Si-stacked photodetection (PD) device.

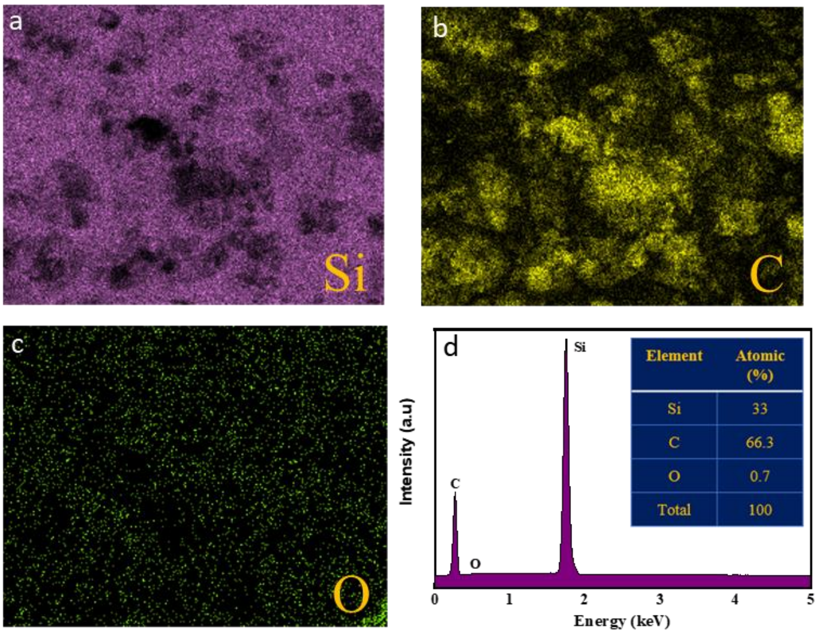

Figure S5. Energy dispersive X-ray spectroscopy (EDS) mapping and images of Si, O, and C elements of graphite film (15 nm thick) transferred on n-type Si substrate.

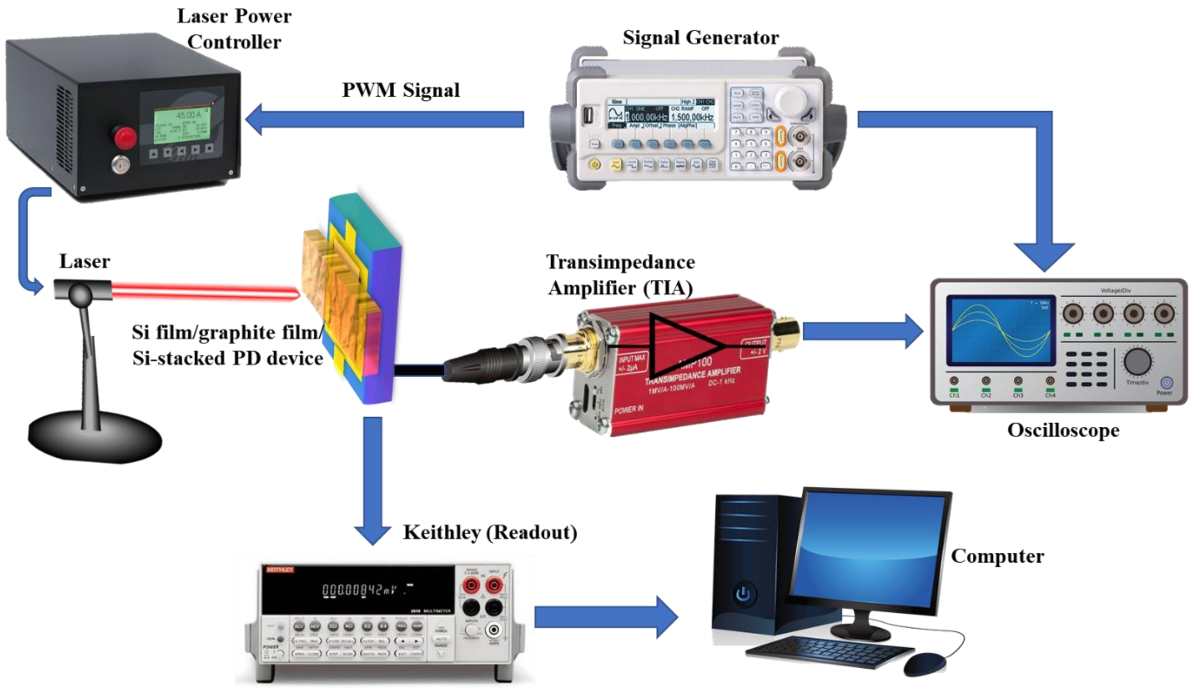

Figure S6. Schematic of the laser system setup for the measurement of characterizations in visible region of p-type Si film/graphite film/n-type Si-stacked Schottky photodiode.
